# Self-sampling to identify pathogens and inflammatory markers in patients with acute sore throat: Feasibility study

**DOI:** 10.3389/fimmu.2022.1016181

**Published:** 2022-10-06

**Authors:** Mark Lown, Elizabeth A. Miles, Helena L. Fisk, Kirsten A. Smith, Ingrid Muller, Emma Maund, Kirsty Rogers, Taeko Becque, Gail Hayward, Michael Moore, Paul Little, Margaret Glogowska, Alastair D. Hay, Beth Stuart, Efi Mantzourani, Chris Butler, Jennifer Bostock, Firoza Davies, Ian Dickerson, Natalie Thompson, Nick Francis

**Affiliations:** ^1^ Primary Care Research Centre, University of Southampton, Southampton, United Kingdom; ^2^ School of Human Development and Health, Faculty of Medicine, University of Southampton, Southampton, United Kingdom; ^3^ Nuffield Department of Primary Care, University of Oxford, Oxford, United Kingdom; ^4^ Centre for Academic Primary Care, National Institute for Health Research (NIHR) School for Primary Care Research, Bristol Medical School: Population Health Sciences, University of Bristol, Bristol, United Kingdom; ^5^ School of Pharmacy and Pharmaceutical Sciences, Cardiff University, Cardiff, United Kingdom; ^6^ Southampton Primary Care Research Centre, Patient and Public Involvement Representative, Southampton, United Kingdom

**Keywords:** sore throat diagnosis, inflammatory markers, swabs, saliva, infection

## Abstract

**Introduction:**

Sore throat is a common reason for overuse of antibiotics. The value of inflammatory or biomarkers in throat swab or saliva samples in predicting benefit from antibiotics is unknown.

**Methods:**

We used the ‘person-based approach’ to develop an online tool to support self-swabbing and recruited adults and children with sore throats through participating general practices and social media. Participants took bacterial and viral swabs and a saliva sponge swab and passive drool sample. Bacterial swabs were cultured for streptococcus (Group A, B, C, F and G). The viral swab and saliva samples were tested using a routine respiratory panel PCR and Covid-19 PCR testing. We used remaining viral swab and saliva sample volume for biomarker analysis using a panel of 13 biomarkers.

**Results:**

We recruited 11 asymptomatic participants and 45 symptomatic participants. From 45 symptomatic participants, bacterial throat swab, viral throat swab, saliva sponge and saliva drool samples were returned by 41/45 (91.1%), 43/45 (95.6%), 43/45 (95.6%) and 43/45 (95.6%) participants respectively. Three saliva sponge and 6 saliva drool samples were of insufficient quantity. Two adult participants had positive bacterial swabs. Six participants had a virus detected from at least one sample (swab or saliva). All of the biomarkers assessed were detectable from all samples where there was sufficient volume for testing. For most biomarkers we found higher concentrations in the saliva samples. Due to low numbers, we were not able to compare biomarker concentrations in those who did and did not have a bacterial pathogen detected. We found no evidence of a difference between biomarker concentrations between the symptomatic and asymptomatic participants but the distributions were wide.

**Conclusions:**

We have demonstrated that it is feasible for patients with sore throat to self-swab and provide saliva samples for pathogen and biomarker analysis. Typical bacterial and viral pathogens were detected but at low prevalence rates. Further work is needed to determine if measuring biomarkers using oropharyngeal samples can help to differentiate between viral and bacterial pathogens in patients classified as medium or high risk using clinical scores, in order to better guide antibiotic prescribing and reduce inappropriate prescriptions.

## Introduction

Acute sore throat is one of the most common reasons for the overuse of antibiotics (1).Throat infections are most commonly caused by viruses but it can be difficult to differentiate between bacterial and viral infections (2). Throat swabs can help guide prescribing but delays in bacteriology results limit their use in general practice and hence they are not recommended for routine use (3).One strategy to reduce antibiotics is to use rapid tests (either alone or in combination with a clinical scoring system) for group A streptococcus to guide antibiotic prescriptions (4). Rapid antigen detection testing (RADT) has a specificity of greater than 95% and sensitivity of 70%–90% for Group A Beta-Haemolytic Streptococcus (GABHS) (5). However, a recent review concluded that although rapid testing for sore throat in primary care probably reduces antibiotic prescription rates by 25%, it may have little or no impact on antibiotic dispensing (4). The National Institute for Health and Care Excellence (NICE) concluded that the use of RADT by general practitioners (GPs) was unlikely to be a cost-effective use of National Health Service (NHS) resources when added to clinical assessment by GPs (3).

One of the problems with testing for GABHS, whether by culture or antigen detection, is that more than 1 in 10 healthy children carry GABHS and up to a quarter of symptomatic children who have GABHS detected are actually only colonised with the organism (6). An approach that may get around this issue, and therefore supplement or replace testing for GABHS, is measuring markers of inflammation from the throat mucosa (7). Bacterial and viral infections produce different inflammatory responses, and molecules associated with these inflammatory responses can be detected using molecular techniques which could potentially be developed into new rapid diagnostic tests. However, although the potential for using biomarkers to differentiate bacterial from viral infections is substantial, (7–9) research into using biomarkers from throat mucosal samples as a tool for guiding antibiotic prescribing for sore throat is at an early stage. We have previously demonstrated that calprotectin, a marker of neutrophil activity, can be measured from throat swab samples and that there is some evidence of an association with streptococcal infection (7).

The COVID-19 pandemic has resulted in an increase in remote consulting in primary healthcare in many countries, with increased experience and acceptance of self testing. In the UK about 90% of primary care consultations were conducted remotely in April 2020 (10). Early in the pandemic, guidance was issued indicating that taking a throat swab was a high-risk procedure for transmission of COVID-19 infection and that the oropharynx should not be examined unless absolutely necessary (11). If biomarkers from throat swabs or saliva are found to be useful in detecting bacterial throat infections, self-collection of these samples would improve the feasibility and safety of such an approach. Taking saliva samples may be more acceptable than throat swabs, especially for children.

There is therefore a need to: 1) evaluate the feasibility of home sampling using throat swabs and saliva sampling, and 2) evaluate the feasibility of using biomarkers from throat swab samples to guide antibiotic prescribing decisions. However, before we can design and conduct an adequately powered study, we need to identify the barriers and facilitators to undertaking research in this setting, and asses the feasibility of conducting a larger study. Our overall aims were therefore to evaluate the feasibility of conducting a study involving self-assessment of the features included in clinical scores used for diagnosing sore throat, and throat self-sampling, and to explore the potential for detecting inflammatory markers for diagnosing sore throat. This will allow us to develop a future study based around a clear understanding of the barriers and facilitators to undertaking this research and determine candidate biomarkers to take forward to the next study. Data on self-assessment of clinical features and self-taken photographs will be reported in a separate publication.

## Methods

First, we set out to develop an online tool to support self-assessment and sampling. This involved using the person-based approach which will be described in more detail in our article describing the self-assessment of clinical features (currently under review). Participants in the development stage (stage 1) were asymptomatic.

### Recruitment

The study was approved by the South West – Cornwall and Plymouth Research Ethics Committee in December 2020 (ref 20/SW/0175). Adults and children with acute sore throat were identified by participating general practices and an out of hours clinic, and directly through advertising. Thirteen participating general practices identified potentially eligible participants during routine consultations and advertised the study on their websites. Potential participants were invited to express an interest through the study website and were then contacted by a member of the study team. Direct advertising was done *via* social media and through established participant recruitment websites. Inclusion criteria were: 1) ongoing sore throat for up to 14 days, 2) able to communicate in English by videoconferencing, 3) aged 17 years or more (adult) or 3-16 years of age with a parent willing to consent, 4) able and willing to comply with study protocol. Exclusion criteria were: 1) any significant disease, disorder, or finding which may significantly increase the risk to the participant, affect their ability to participate or impair interpretation of the study data, 2) participation in another clinical trial within the last 3 months.

We also recruited 11 asymptomatic (stage 1) participants who helped develop test and refine the study website. The stage one participants were asked to follow the same testing regime. Results from their samples are presented for comparison.

### Consent and data collection

Participants consented remotely/online with parents asked to consent on behalf of their children (who provided assent). Using an online questionnaire, participants entered baseline data including demographics (age, sex, ethnicity), symptoms (presence, duration, and severity) and whether they had been prescribed antibiotics for their sore throat. A test kit including swabs and saliva collection kits was sent to each participant’s home by next day delivery.

Participants were observed by a member of the study team whilst they made a clinical assessment of their throat and taking throat samples *via* online video call. Assessments included the criteria used in the FeverPAIN and Centor scores and photographs of their throat. The results of this self-assessment aspect of the study will be reported separately. Samples requested were two throat swabs (viral and bacterial), one saliva drool pot sample (passive drool into a universal container), and one saliva sponge sample (different sponges for adults and children (https://salimetrics.com/saliva-collection-from-infants-and-small-children/). Participants sent the biological samples directly to the laboratory using packages with prepaid postage.

### Sample size

This was a feasibility study, so the sample size was based on an estimate of the precision for key feasibility outcomes. 40 participants would allow us to estimate a proportion (such as participation rate) of 50% with a 95% confidence interval of +/- 11% (50% is the most conservative estimate to use). We aimed to recruit both younger children (3–5) and older children > 5 years as we anticipated there may be specific challenges for parents assessing and testing young children’s sore throats.

### Laboratory sample analyses

The samples were used to evaluate the feasibility of determining the presence or absence of streptococcus in addition to common viral pathogens and Covid-19, and to analyse biomarker concentrations. Throat culture samples were processed on arrival to the laboratory in accordance with standard clinical practice and cultured for streptococcus (Group A, B, C, F and G). The absorbent materials were compressed to extract as much volume as possible. The first 0.5 ml for each of the 3 samples (swab, drool and absorbent sponge) was aliquoted into a vial for viral analysis and the remaining volume aliquoted into another vial for biomarker analysis. All samples were frozen at -80 degrees C and batch analysed at the end of the study. Viral analysis consisted of a routine respiratory panel PCR (Influenza A, Influenza B, RSV A/B (no distinction), Metapneumovirus, Parainfluenza 1, 2 and 3, Rhinovirus, Adenovirus) and Covid-19 PCR testing. In terms of extraction, we used the QIagen Symphony SP instrument; DSP Virus/Pathogen Mini Kit; Complex off-board lysis 200 µL protocol (200µL of sample input) and 85µL elution volume. We used Qiagen Agility for post elution and ABI 7500 for PCR and analysis. Low level PCRs were reported for any value of a cycle threshold of 35 or higher. Positive results were reported for any value of a cycle threshold below 35. Inhibitory specimens were reported for late or negative cycle threshold values, for the control assay, whilst being negative for target assays that were performed alongside.

Saliva samples from the sponge swab or passive drool collection and diluent from the viral swab were defrosted. Samples were centrifuged to remove debris prior to analysis.

Biomarkers were measured by enzyme-linked immunoassay or multiplex assays. An enzyme-linked immunoassay was used to measure calprotectin (Invitrogen, ThermoFisher Scientific, Paisley, UK).

Interferon-alpha, interferon beta, CXCL10/IP-10, matrix-metalloproteinase (MMP)-8 and MMP-9 were measured using Luminex multiplex kits (R&D Systems, Bio-techne, Abingdon, UK) following the manufacturer’s instructions. Interferon-gamma, interleukin (IL)-1β, IL-6, IL-17A, IL-21, lipocalin-2/NGAL, and neutrophil elastase-2 (ELA2) were measured using Milliplex multiplex kits following the manufacturer’s instructions (Merck Life Science UK Limited, Dorset, UK). All multiplex kit plates were read using a Bio plex 200 plate reader (Bio-Rad, Watford, UK).

Lower limits of detection (pg/ml) were: Calprotectin (35.0), interferon alpha (0.26), interferon beta (0.48), CXCL10/IP-10 (1.18), MMP-8 (34.2), MMP-9 (13.6), interferon gamma (1.8), IL-1β (2.1), IL-6 (1.7), IL-17A (2.1), IL-21 (2.0), lipocalin-2 (15.0), neutrophil elastase-2 (3.0).

## Results

**Figure 1:**
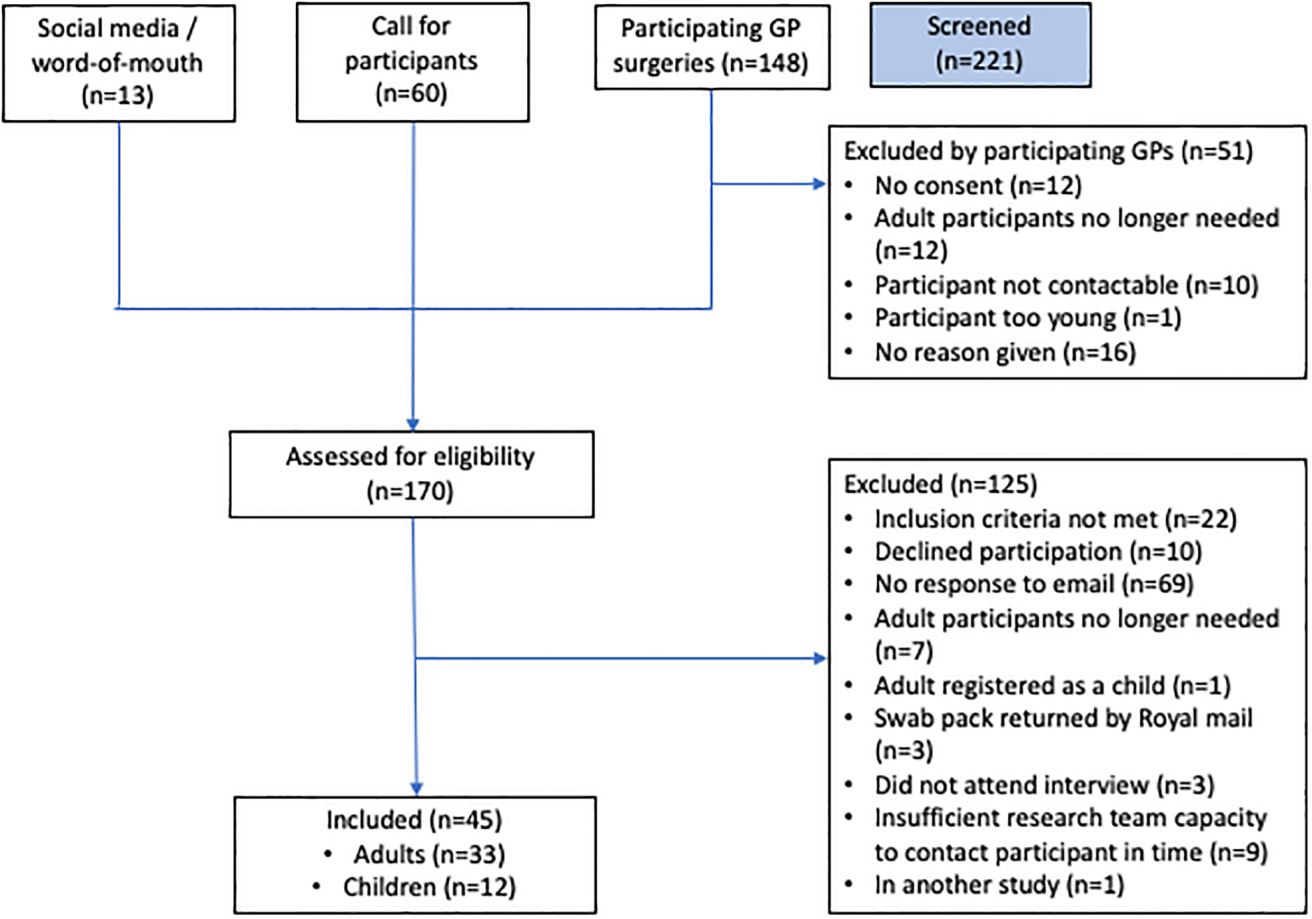
Recruitment flow diagram.

A total of 45 participants (33 adult and 12 children) with sore throat took part in the study (**Figure 1**). In addition, 11 asymptomatic participants (7 adults, 3 children aged 6-15 and 1 child aged between 3 and 5) were involved in Stage 1 (intervention development) and their throat biomarker data are used for comparison.

One stage 1 participant had missing data on gender (25%) and 1 stage 2 adult (3.0%) had missing data on ethnicity but there were no other missing demographic data (**Table 1**). Stage 2 adult participants were mostly female (69.7%), young (median age 28 years, IQR 21 to 38 years) and white (75.0%), with ethnic mix broadly in line with the UK population (given the small numbers). Stage 2 child participants were evenly split between sex (50.0% female), with a median age of 9 years (IQR 5.5 to 12.5) and mostly white (66.6%). The self-reported clinical characteristics of the stage 2 participants are shown in **Table 2**.

**Table 1:** Characteristics of stage 1 and stage 2 participants.

	Stage 1 adult participant (N = 7)	Stage 1 child participant (N = 4)	Stage 2 adult participant (N = 33)	Stage 2 child participant (N = 12)
Gender – female (n, %)	4 (57.1)	2 (50.0)	23 (69.7)	6 (50.0)
Missing (n, %)	0 (0.0%)	1 (25.0%)	0 (0.0%)	0 (0.0%)
Age, years (median, IQR)	31 (26, 34)	9 (4.5, 13.5)	28 (21, 38)	9 (5.5, 12.5)
Missing (n, %)	0 (0.0%)	0 (0.0%)	0 (0.0%)	0 (0.0%)
Ethnicity (n, %)				
White	5 (71.4)	4 (100.0)	24 (75.0)	8 (66.6)
Asian	2 (28.6)	0 (0.0)	5 (15.6)	2 (16.7)
Black	0 (0)	0 (0.0)	1 (3.1)	2 (16.7)
Mixed	0 (0)	0 (0.0)	1 (3.1)	0 (0.0)
Other	0 (0)	0 (0.0)	1 (3.1)	0 (0.0)
Missing (n, %)	0 (0%)	0 (0.0%)	1 (3.0%)	0 (0.0%)

**Table 2:** Clinical characteristics*.

	N	Adult	N	Child
Severity score^†^ (median, IQR)	33	6 (4, 7)	12	8 (7, 8)
Sore throat started less than 3 days ago (n, %)	33	10 (30.3)	12	3 (25.0)
Fever (n, %)	33	7 (21.2)	12	4 (33.3)
Persistent cough (n, %)	33	3 (9.1)	12	3 (25.0)
Runny/blocked nose/sneeze (n, %)	33	14 (44.2)	12	4 (33.3)
Throat very swollen/red (n, %)	33		12	
Yes		15 (45.5)		9 (75.0)
No		10 (30.3)		2 (16.7)
Not sure		8 (24.2)		1 (8.3)
Yellow/white spots on throat (n, %)	33	11 (33.3)	11	6 (54.6)
Painful lymph nodes (n, %)	33		11	
Yes		14 (42.4)		5 (45.5)
No		12 (36.4)		1 (9.1)
Not sure		7 (21.2)		5 (45.5)
FeverPAIN score (n, %)	33		12	
0-1		7 (21.2)		2 (16.7)
2-3		17 (51.5)		7 (58.3)
4+		9 (27.3)		3 (25.0)
Centor score (n, %)	33		12	
0-1		14 (42.4)		3 (25.0)
2-3		16 (48.5)		9 (75.0)
4		3 (9.1)		0 (0.0)
Antibiotics (n, %)	33	7 (21.2)	12	7 (58.3)

*Self-assessed.

†Severity score on a scale of 1 to 10 with 10 being the most severe.

### Self-sampling

Stage 2 sample receipt and processing is summarised in **Table 3**. Bacterial throat swabs were returned by 41/45 (91.1%) symptomatic participants. One swab was unlabelled and not processed. The 5 participants who did not return swabs were all adults. Viral throat swabs were returned by 43/45 (95.6%) participants. 42/45 (93.3%) samples were processed by the laboratory. One older child sample was not processed by the laboratory in error. Sponge saliva samples were returned by 43/45 (95.6%) of participants and 3 adult samples were of insufficient quantity. Passive drool **s**aliva samples were received from 43/45 (95.6%) of participants. Six (adult) samples were of insufficient quantity and were not processed by the laboratory. In addition, the 11 stage 1 participants provided all samples except for 3 older children who did not provide a saliva drool sample).

**Table 3:** Stage 2 samples/tests received and processed for bacterial and viral analysis.

Sample type	Received at laboratory	Processed by laboratory	Missing samples by age group
Bacterial swab	41/45 (88.9%)	40/45 (88.9%) (1 unlabelled swab)	5 adults
Viral swab	43/45 (95.6%)	42/45 (93.3%) 1 swab was not processed by laboratory (laboratory error)	1 adult, 1 young child (samples not returned), 1 adult not processed by laboratory
Saliva sponge	43/45 (95.6%)	40/45 (88.9%) 3 insufficient samples	1 adult, 1 young child (not returned) 3 adults – insufficient sample
Saliva drool	43/45 (95.6%)	37/45 (82.2%%) 6 insufficient samples	1 adult, 1 young child (not returned) (6 adults, - insufficient)

### Microbiology results

Two adult participants had positive bacterial swabs (one with a moderate growth of streptococcus G and one with a heavy growth of streptococcus B). Six participants had viral pathology detected from at least one positive sample (swab or saliva) and eight participants had inconclusive (neither positive nor negative) for adenovirus in all samples received (**Table 4**).

**Table 4:** Stage 2 virology results.

Virus	Age group	Throat swab	Saliva sponge	Saliva drool
Rhinovirus	Child	Positive	Negative	Low level
Influenza A	Young adult	Negative	Low level	Low level
Influenza A	Young adult	Negative	Low level	Negative
Influenza A	Adult	Low level	Negative	Negative
Rhinovirus	Young adult	Positive	Low level	Low level
Rhinovirus	Young adult	Positive	Low level	Negative

The proportion of stage 1 + stage 2 samples with sufficient volume for biomarker analysis by sample type are listed in **Table 5**.

**Table 5:** Proportion of samples received by laboratory with sufficient volume for biomarker analysis by sample type.

Sample type	Stage 1 – asymptomatic analysable samples	Stage 2 – symptomatic analysable samples
Viral swab	11/11 (100.0%)	39/45 (86.7%) (30/33 (90.9%) adults) (0/3 (0.0%) younger children) (9/9 (100.0%) older children)
Saliva sponge	11/11 (100.0%)	33/45 (73.3%) (25/33 (75.8%) adults) (1/3 (33.3%) younger children) (8/9 (88.9%) older children)
Saliva drool	5/11 (45.4%) (5/7 (71.4%) adults) (0/3 (0.0%) older children) (0/1 (0.0%) younger children)	23/45 (51.1%) (16/33 (48.5%) adults) (0/3 (0.0%) younger children) (7/9 (77.8%) older children)

All of the biomarkers assessed were detectable from all samples where there was sufficient volume for testing. For most biomarkers we found higher concentrations in the saliva samples (**Table 6**). Box plots of the biomarker values are presented in **Supplementary Figure 1**.

**Table 6:** Biomarker concentrations detected in throat swabs and saliva samples.

Biomarker	Throat swab			Saliva sponge			Saliva drool pot		
	Asymp -Min, Max, median (n)	Symp-Min, Max, median (n)	P Mann Whitney	Asymp -Min, Max, median (n)	Symp-Min, Max, median (n)	P Mann Whitney	Asymp -Min, Max, median (n)	Symp-Min, Max, median (n)	P Mann Whitney
Calprotectin (pg/ml)	160.0, 6427.2, 1806.3 (11)	160.0, 35192.7, 3430.1 (39)	0.81	513.9, 53465.3, 4252.2 (11)	160.0, 3.52 x 10^8^, 4730.5 (33)	0.84	4935.5, 1.76 x 10^8^, 27097.2 (5)	1368.1, 55866.1, 5143.7 (23)	0.04
MMP8 (pg/ml)	134.67, 5337.02, 485.28 (10)	102.60, 73228.40, 900.30 (38)	0.35	597.08, 53377.10, 7245,74 (11)	615.72, 39971.70, 6263.86 (33)	0.54	13186.30, 85996.80, 18358.15 (4)	126.92, 122713.00, 13262.6 (24)	0.26
MMP9 (pg/ml)	236.43, 4035.02, 975.23 (10)	108.75, 33367.10, 1463.23 (38)	0.53	384.94, 15143.30, 3200.26 (11)	544.35, 14143.50, 4300.93 (33)	0.58	5286.25, 12655.60, 7288.20 (4)	96.02, 16240.50, 5228.25 (24)	0.32
CXCL10 (pg/ml)	0.14, 121.52, 5.82 (11)	0.14, 331.84, 0.95 (36)	0.10	0.14,14.33, 0.93 (11)	0.14,75.83, 7.53 (31)	0.31	0.14,22.21, 0.14 (5)	0.14, 127.99, 0.21 (22)	0.66
IFN b (pg/ml)	0.86,3.20, 0.86 (11)	0.86,10.90, 1.71, (36)	0.31	0.86,10.01, 0.86 (11)	0.86,25.90, 0.86(31)	0.83	0.86,3.20, 0.86 (5)	0.86,24.34, 0.86 (22)	0.73
IFN a (pg/ml)	0.15,4.58, 1.98 (11)	0.15,15.28, 2.37, (36)	0.52	0.15,10.71, 0.78 (11)	0.15,8.84, 0.78 (31)	0.93	0.155.37, 0.15(5)	0.1514.98, 1.61 (22)	0.45
IFN g (pg/ml)	0.01,44.69, 1.23 (10)	0.00, 28.87, 1.00 (38)	0.59	0.01,1.46, 0.01 (8)	0.01,14.31, 2.01 (32)	0.04	0.01,0.01,0.01 (2)	0.01,36.34, 0.96 (23)	0.17
NGAL (pg/ml)	129.00, 21691.60, 2579.06 (11)	28.69, 68311.20, 2830.32 (39)	0.60	9410.52, 97010.40, 27569.40 (11)	14.50, 51948.30, 10986.7 (33)	0.01	30675.60, 8260000.00, 488 23.80(5)	14.16, 239399.00, 15346 (24)	0.06
Elas2 (pg/ml)	32.12 14362.20, 909.50 (11)	7.03 147210.00, 1772.93(39)	0.77	62.05 50368.20, 10627.10 (11)	11.66 58211.90, 2782.35 (33)	0.11	17504.80, 125905.00, 31106.40 (5)	11.66 700515.00, 11413.06 (24)	0.17
IL17 (pg/ml)	0.131.99, 0.13(10)	0.13, 25.81, 0.13 (38)	0.91	0.13,9.92, 0.13 (8)	0.1324.17, 0.13(32)	0.81	0.13,0.13, 0.13 (2)	0.13,0.13, 28.41 (23)	0.47
IL1b (pg/ml)	2.27, 230.17, 12.70 (10)	0.05, 110.38, 4.77 (38)	0.06	0.05,25.27, 2.51 (8)	0.05,75.69, 6.10 (32)	0.19	0.05,1.57, 0.81(2)	0.05, 490.06, 7.13 (23)	0.09
IL21 (pg/ml)	0.77,22.24, 4.56 (10)	0.39,45.09, 1.35 (38)	0.03	0.3915.38, 1.93 (8)	0.39,37.08, 3.14 (32)	0.31	0.39,6.54, 3.47 (2)	0.3931.96, 2.50 (23)	0.61
IL6 (pg/ml)	0.288.76, 2.05 (10)	0.28,39.15, 2.19 (38)	0.71	0.2810.77, 0.42 (8)	0.2876.13, 2.86 (32)	0.24	0.280.28, 0.28(2)	0.28 289.56, 3.69 (23)	0.12

As we only had two participants in whom we detected a potential bacterial throat pathogen we were not able to compare biomarker concentrations in those who did and did not have a bacterial pathogen. (Group B streptococcus is not usually considered pathogenic in acute sore throat although it was isolated from the throats of 49/1,110 patients with pharyngitis in a study reported in 1979 that suggested an association with positive clinical findings) (12). We found no strong evidence of a difference between biomarker concentrations in those with and without sore throat (**Table 6**), but the distributions were wide and we were not powered to detect clinically important differences.

## Discussion

Our study has demonstrated that it is feasible and acceptable for patients to collect swabs and saliva samples in adult and child participants with sore throat with over 90% of participants returning each sample type (bacterial swab, viral swab, saliva drool and saliva absorbent sponge). The majority of samples were analysed by the laboratory although 17.8% of the saliva pot samples had insufficient volume for viral analysis. Another study using saliva drool methods reported high proportion of samples with insufficient volume (29.2%) (13).

In this study with a small number of participants, two participants had positive streptococcus swabs (group B and G). Group A beta-haemolytic streptococcus (GABHS), the most common bacterial etiology, accounts for 15 to 30 percent of cases of acute pharyngitis in children and 5 to 20 percent in adults (14). Non–group A beta-haemolytic streptococci (groups C and G) also can cause acute pharyngitis. The proportion of participants with detected streptococcus was very low and there are several potential reasons for this. Firstly, COVID-19 prevention measures were shown to have dramatically reduced the prevalence of streptococcus (15) and Public Health England data showed a reduction in GP coded pharyngitis cases in our recruitment period compared with pre-pandemic levels (16). In addition, a significant proportion of participants were recruited online and *via* social media who may have had milder illness than those presenting to GP surgeries and less-likely to have had a bacterial sore throat. Only 15/45 (33%) of stage 2 participants reported being prescribed antibiotics for their sore throat and in those stage 2 participants with clinician-assessed FeverPAIN scores (using throat photographs taken by participants) (n=18), only 10% recommended immediate antibiotic prescriptions suggesting a low prevalence of bacterial infections. Sub-optimal swabbing technique may have impacted on the detection rate, but self-swabbing without training in lay individuals has been shown to be accurate for detecting COVID-19 (17) and some participants in this study had prior experience of self-swabbing for COVID-19. In addition, swabbing of other areas of the mouth has been shown to be accurate for streptococcus detection (18). Finally, our low detection rate may have resulted from pathogens becoming non-viable during transit. The median time between sample collection and analysis was 3 days, compared with an average of 2.7 days in another study comparing parent-collected with nurse-collected nasal swabs where the sensitivity for Streptococcus pyogenes detection in parent-collected swabs was 87.5% using nurse-collected swab as the reference (19). More recently, evidence has shown that Fusobacterium are commonly detected in patients with acute pharyngitis (20) and future evaluation of cytokines should consider this.

Influenza A and rhinovirus were detected in 6 stage 2 participants and are common causes of viral sore throat. No participants in this study were found to be co-infected with multiple pathogens. The proportion of participants with detected influenza A (3/45 = 6.7%) were similar to those detected in another study carried out in 83 university students pre-pandemic in Canada (9.6%) However, levels of other pathogens including rhinovirus were lower (3/45 = 6.7% vs 26.5%) (21). Although we did not assess the accuracy of self-swabbing versus clinician swabbing, a recent systematic review and meta-analysis of upper airway swab collection (for detection of viral and bacterial pathogens by individuals or caregivers compared to health care workers) found a sensitivity of 91% and specificity of 98% and good acceptability (22). The review included pharyngeal and nasal swabs, pathogens included streptococcus and viral panels and studies included child and adult participants. The review concluded that swabs by health care workers should not be automatically assumed to be superior to swabs collected by individuals or their caregivers.

Interestingly, no single method (swab, sponge or drool) detected the viruses in all cases in this study and further work is needed to determine the most accurate and acceptable method of sampling. A recent diagnostic study in children aged under 18 found good accuracy for saliva drool or sponge samples compared with a combined reference standard defined as detection of a viral pathogen in at least one sample (nasopharyngeal swab, oropharyngeal swab or saliva sample) with a PCR based respiratory panel of 21 pathogens (23). The sensitivity in nasopharyngeal swabs was 93% (95% confidence interval [CI]: 78%-98%), in oropharyngeal swabs 79% (95% CI: 60%-90%), in saliva overall 76% (95% CI: 58%-88%) and in 18 saliva samples collected with drooling or sponges, 94% (95% CI: 74%-99%). The authors concluded that saliva could be a relevant specimen alternative to throat swabs (23). The COVID-19 pandemic has further led to interest in evaluating simple, non-invasive sample collection methods that are acceptable for repeat testing that can be carried out by individuals themselves. Saliva testing has been shown to be similarly sensitive and less costly alternative to nasopharyngeal swabs for covid testing (24).

In this study, we were able to detect saliva levels of 13 different biomarkers using throat swabs and saliva drool and saliva sponge collection methods and in general the salivary concentrations were higher than the throat swab concentrations. We also found no evidence of a difference between symptomatic and asymptomatic participants although this was not a primary objective and the clinical utility of biomarkers would be in differentiating bacterial and viral infections. As only two participants were found to have bacterial pathogens, we did not perform any further analysis of biomarkers to discriminate bacterial versus non-bacterial infection. Previous work has shown that calprotectin measured by throat swabs is high in patients with sore throat likely to be caused by streptococcal infection (7). Calprotectin has been shown to be elevated in the serum of patients with acute respiratory infections and to aid discrimination between bacterial and viral infections (25). Saliva calprotectin has also been shown to correlate strongly with serum calprotectin in a small study of hospitalised children with community acquired pneumonia (26). We were able to measure calprotectin in symptomatic/stage 2 participants in 86.7%, 73.3%, and 51.1% *via* throat swab, saliva sponge and saliva drool respectively.

Interestingly, saliva calprotectin has been shown to correlate with systemic inflammation but not periodontal parameters (in children with cystic fibrosis) and could have a potential role in determining bacterial colonisation rather than reflecting concomitant gingival inflammation (27). Salivary biomarkers have also been shown to correlate with serum counterparts in COPD patients and to increase during exacerbations. Further work is needed to determine if other biomarkers detected using oropharyngeal samples have diagnostic potential in bacterial throat infections. It should be noted that salivary biomarkers such as interleukins, growth factors, enzymes, and other biomolecules can be elevated due to other diseases of the oral cavity including oral lichen planus and peridontitis (28). Other factors such as stress may cause rises in salivary biomarkers (29).

### Limitations

Our findings are limited by small numbers of participants including small numbers of children. Low rates of detected pathogens limited our analysis and we did not compare self-swabs with swabs collected by health care practitioners so cannot be sure that the self-swabbing techniques were adequate. We asked participants to provide two swab and two saliva samples sequentially which may have impacted on the volume of saliva collected. We did not provide specific guidance on collecting saliva samples with respect to time of day, rinsing the mouth or strict guidance on last time of consumption of food. We did not assess the stability of the biomarker samples with respect to transit time and storage at room temperature before freezing.

## Conclusion

We have demonstrated that it is feasible for patients with sore throat to self-swab and provide saliva samples (passive drool and sponge) for pathogen and biomarker analysis. Typical bacterial and viral pathogens (streptococcus, influenza A and rhinovirus) were detected in some of the participants albeit at low prevalence rates which could have been affected by the COVID-19 pandemic or loss of viability during specimen transit. Further work is required to determine if self-swabbing is accurate. We were able to detect 13 measured biomarkers from the swabs and saliva samples but further work is needed to determine if measuring biomarkers using oropharyngeal samples can help to differentiate between viral and bacterial pathogens in patients classified as medium or high risk using clinical scores, in order to better guide antibiotic prescribing and reduce inappropriate prescriptions.

## Data availability statement

The raw data supporting the conclusions of this article will be made available by the authors, without undue reservation.

## Ethics statement

This study involving human participants were reviewed and approved by South West – Cornwall and Plymouth Research Ethics Committee in December 2020 (ref 20/SW/0175). Written informed consent to participate in this study was provided by the participants’ legal guardian/next of kin.

## Author contributions

NF and ML conceived and designed the study. All authors contributed to the study materials and management. ML wrote the first draft of the manuscript. All authors contributed to revising the manuscript and approved the final version.

## Funding

This study/project is funded by the National Institute for Health and Care Research (NIHR) School for Primary Care Research (project reference 463). The views expressed are those of the author(s) and not necessarily those of the NIHR or the Department of Health and Social Care.

## Conflict of interest

The authors declare that the research was conducted in the absence of any commercial or financial relationships that could be construed as a potential conflict of interest.

## Publisher’s note

All claims expressed in this article are solely those of the authors and do not necessarily represent those of their affiliated organizations, or those of the publisher, the editors and the reviewers. Any product that may be evaluated in this article, or claim that may be made by its manufacturer, is not guaranteed or endorsed by the publisher.
