# Supplementary material for: Self-sampling to identify pathogens and inflammatory markers in patients with acute sore throat: Feasibility study
Source: Front Immunol. 2022 Oct 6;13:1016181. doi: 10.3389/fimmu.2022.1016181 (PMC9582425; doi:10.3389/fimmu.2022.1016181)
Supplement: Supplementary file 1 [file DataSheet_1.docx]

**Supplementary Figure 1 – Box plots of biomarker analysis by sample type**
